# Draft Genome Sequence of *Alteromonas* sp. Strain RKMC-009, Isolated from *Xestospongia muta* via *In Situ* Culturing Using an Isolation Chip Diffusion Chamber

**DOI:** 10.1128/MRA.00508-19

**Published:** 2019-06-20

**Authors:** Logan W. MacIntyre, Bradley A. Haltli, Russell G. Kerr

**Affiliations:** a Department of Biomedical Sciences, University of Prince Edward Island, Charlottetown, Prince Edward Island, Canada; b Department of Chemistry, University of Prince Edward Island, Charlottetown, Prince Edward Island, Canada; c Nautilus Biosciences, Regis and Joan Duffy Research Centre, Charlottetown, Prince Edward Island, Canada; University of Rochester School of Medicine and Dentistry

## Abstract

We report the draft whole-genome sequence of Alteromonas sp. strain RKMC-009, which was isolated *in situ* from the sponge Xestospongia muta in San Salvador, The Bahamas, using an isolation chip (ichip). Automated biosynthetic gene cluster analysis using antiSMASH 4.0 predicted the presence of 22 biosynthetic gene clusters.

## ANNOUNCEMENT

As part of ongoing work in our natural products research group to discover new microbial taxa from marine invertebrates, we implemented an isolation chip (ichip) for *in situ* bacterial isolation from the giant barrel sponge, Xestospongia muta (1). Samples of X. muta were collected from San Salvador, The Bahamas, and ichips were fabricated at the University of Prince Edward Island. Alteromonas sp. strain RKMC-009 was isolated from an ichip implanted in *X. muta* and remains of high interest to our research group for the production of natural products. To enable the identification of natural product biosynthetic gene clusters (BGCs) in strain RKMC-009, we obtained a draft whole-genome sequence.

Strain RKMC-009 was cultivated for 3 days in ISP2 medium (1% malt extract, 0.4% dextrose, 0.4% yeast extract) supplemented with 1.8% Instant Ocean (Spectrum Brands) at 30°C with orbital shaking (200 rpm). Genomic DNA was prepared using the DNeasy UltraClean microbial DNA isolation kit, according to the instructions provided by the manufacturer (Qiagen). Library construction and DNA sequencing were carried out by the McGill University and Génome Québec Innovation Centre (Montréal, Canada) using a standard PacBio protocol for 20-kb libraries and a PacBio RS II single-molecule real-time platform with P4-C2 chemistry (Pacific Biosciences, USA). *De novo* assembly was performed by the sequencing facility using the Hierarchical Genome Assembly Process (HGAP2)/Quiver protocol in SMRT Portal version 2.3.0 (2). A minimum seed length achieving 30× sequence coverage was chosen for assembly. Overall, 91,263 reads were assembled into a draft genome sequence accounting for 5,060,348 bp across 2 contigs (*N*_50_, 4,959,037 bp) at 254-fold coverage. Automated circularization was performed using Circlator with default parameters (3). Contig 1 corresponds to a circular chromosome (4,959,037 bp) with a G+C content of 48.7%. Circlator did not circularize contig 2 (101,311 bp); however, BLASTN analysis of the ends of the contig identified a 23,881-bp overlapping region (4). We manually circularized the plasmid sequence by joining nucleotide 1 to nucleotide 77430 and confirmed the proposed circularization by sequencing PCR products spanning the circularization points and regions with mismatched nucleotides within the overlap. The results of these analyses helped us determine the sequence of a 77,430-bp circular plasmid with a G+C content of 42.8%. Both contigs were annotated using the NCBI Prokaryotic Genome Annotation Pipeline (PGAP), which predicted 4,289 protein-coding DNA sequences (CDSs), 15 rRNAs, and 60 tRNAs for the chromosome and 67 CDSs and no RNAs for the plasmid (5). The type strain most closely related to RKMC-009 based on 16S rRNA gene sequence analysis (1,543 bp; nucleotides 32656 to 34198) using the EzBioCloud tool is Alteromonas aestuariivivens JDTF-113 (GenBank accession number KY497472), with 98.15% sequence similarity (6). A whole-genome average nucleotide identity (wgANI) comparison between RKMC-009 and 11 closely related *Alteromonas* sp. genomes using JSpeciesWS indicated that the highest identity was to Alteromonas confluentis KCTC 42603^T^ (75.65%) (7). The low 16S rRNA gene sequence and wgANI identities with validly published *Alteromonas* species suggest that RKMC-009 may represent a new *Alteromonas* species (8–10).

Automated analysis of secondary metabolite biosynthetic gene clusters (BGCs) was carried out using antiSMASH version 4.0 (11). The chromosome was predicted to contain 22 BGCs, and none were detected within the plasmid. Three BGCs were assigned to a specific secondary metabolite class (aryl polyene, bacteriocin, and lasso peptide), three were classified as putative fatty acid biosynthetic pathways, and the remainder were unclassified putative BGCs as identified by the ClusterFinder algorithm.

### Data availability.

This draft whole-genome sequence has been deposited in GenBank under the accession numbers CP031010 and CP032914. The versions described in this paper are the first and third versions, CP031010.1 and CP032914.3, respectively. Raw sequence reads have been deposited into the Sequence Read Archive under the accession number SRR8209716.
